# Screening for frailty among older emergency department visitors: Validation of the new FRESH-screening instrument

**DOI:** 10.1186/s12873-016-0087-0

**Published:** 2016-07-22

**Authors:** Eklund Kajsa, Wilhelmson Katarina, Landahl Sten, Ivanoff-Dahlin Synneve

**Affiliations:** Vårdalinstitutet, The Swedish Institute for Health Sciences, University of Gothenburg, Lund, Sweden; Department of Health and Rehabilitation, The Sahlgrenska Academy at University of Gothenburg, Gothenburg, Sweden; Department of Clinical Neuroscience and Rehabilitation, University of Gothenburg, Center of Aging and Health-AGECAP, Gothenburg, Sweden; Institute Neuroscience and physiology, Gothenburg University, Gothenburg, Sweden

**Keywords:** Frailty, Sensitivity, Specificity

## Abstract

**Background:**

The identification of frail older persons in different health care settings is widely seen as an important step in improving the healthcare system. Screening at an emergency department (ED) should be handled in just a few minutes without the use of tests or measurements. The FRESH-screening was developed for this purpose. This study’s aim was to evaluate the FRESH-screening and its construct validity; also assessed were the sensitivity, specificity, and predictive values for frailty screening.

**Methods:**

The study had a cross-sectional design. A total of 161 elderly people who sought care at the emergency department at Mölndal Hospital were included. Inclusion criteria were ages ≥80 years or ages 65–79 with at least one chronic disease and dependence in at least one daily living activity. Sensitivity, specificity, and predictive values were calculated to describe the accuracy of the FRESH-screening in identifying those with frailty, as assessed by eight frailty indicators. Sensitivity and specificity were both set at a minimum of 80 %, and a percentage sum ≥150 of the sensitivity and positive prediction was considered a measure of excellent value.

**Result:**

Both sensitivity and specificity were high (81 % and 80 %, respectively) when comparing the four questions of the FRESH-screening against the eight frailty indicators. The percentage sum of sensitivity and positive prediction was 173 (81 % + 92 %), thus exceeding the 150 cutoff.

**Conclusion:**

This study shows the FRESH-screening to be of excellent clinical value. Additionally, the clinical experience is that the instrument is simple and rapid to use, takes only a few minutes to administer, and requires minimal energy input by older persons.

## Background

The identification of frail older persons in different health care settings is seen as a critical step in improving the healthcare system in the Western world [1, 2]. Comprehensive Geriatric Assessment (CGA) is widely used and accepted as an important component in the evaluation of older persons care needs. CGA involves a multidimensional team approach assessing medical, functional, psychosocial, and environmental needs [3]. However, this approach is time consuming, especially in acute situations. Through the use of a CGA screening instrument, health personnel can focus their efforts on the older persons who have greater healthcare and rehabilitation needs.

There is still no unanimously accepted definition of frailty [4]. However, there are generally two primary definitions currently in use: one based on physical phenotype [5], and one based on broader parameters that include various social and psychological components [6]. A strong consensus among researchers does exist that frailty is characterized by decreased reserves and diminished resistance to stressors [6]; though no agreement has been made on which biomarkers should be included in such an assessment [4]. Commonly, the physical phenotype includes functional frailty indicators such as weakness, fatigue, weight loss, low physical activity, poor balance, and slow gait speed [7]. Visual impairment and impaired cognition have also been highlighted as frailty indicators, based on their impact on morbidity and disability [8, 9]. Recently, a consensus group consisting of delegates from six major international, European, and US societies created major consensus points of physical frailty. Frailty was subsequently defined as “a medical syndrome with multiple causes characterized by diminished strength, endurance, and reduced physiological capacity that increases an individual’s vulnerability for developing increased dependency and/or death” [10].

Depending on which definition of frailty a researcher upholds, prevalence rates of frailty vary. A recent systematic review by Collard et al. [11] showed that the prevalence of frailty by physical phenotype among community-living persons aged 65 years and older ranges from 4 % to 17 %. When the broader frailty definition is used, however, prevalence ranges from 4.2 % to 59.1 %. Collard et al. [11] also reported that frailty prevalence is significantly higher in the 80+ age group. Older frail persons account for the highest healthcare costs in developed countries [12], and frailty has been found to have a higher impact on activities of daily living (ADL) dependence than on morbidity [13]. It is thus imperative to find variables that predict risks of developing frailty and identify persons who could benefit from preventive interventions. According to the abovementioned consensus group, simple screening tests for frailty should be short and valid [10]. The FRAIL scale has been named as one such viable screening test, but it has only been validated for African-Americans aged 49–65 years [14]. Several international screening instruments exist, but few of them are short and easy to use; they also have yet to be validated for ages 75 and above [15], so there is still need for a validated frailty screening instrument.

Ideal screening instruments should have a high sensitivity, ensuring accurate identification of those in need of further care. A high specificity is also important to limit incorrectly identified persons [16]. Instruments should be clinically friendly, or easily accepted in clinical settings by both the older person and the healthcare staff [17]. During the pilot study, “Continuum of care for frail elderly people,” [18] the need for a clinical friendly screening instrument was recognized. Since recruitment was performed at an emergency department the requirements were that it would identify frail older persons in just a few minutes without the use of tests or measurements. Based on these requirements’, a short screening instrument was developed. Data from an earlier study targeting pre-frail older persons [19] guided the identification of simple questions indicating early signs of frailty. Four identified questions, and an additional question of having had three or more visits to the emergency department during the last twelve months constituted the screening instrument. Answering “yes” on two or more of the questions indicated risk of frailty. The screening instrument was considered clinically friendly by all involved parties but still needed to be validated. Accordingly, the aim of this study was to evaluate the questions and construct validity of the short screening instrument; sensitivity, specificity, and predictive value to screen for frailty were also assessed.

## Methods

### Study design

This cross-sectional study composed one part of the broader research program called “Continuum of care for frail elderly people” (http://neurophys.gu.se/sektioner/halsa-och-rehabilitering/forskning/fresh).

The level of inter-instrument agreement between scales refers to construct validity and was determined by the agreement between frailty indicators and the questions in the screening instrument assessed at baseline. Sensitivity, specificity, predictive value, and the identification of frailty were calculated on the basis of Fried’s classical frailty indicators [7] with the use of validated instruments and questionnaires [18].

### Sample and setting

The study group included 161 elderly people who sought care at the emergency department at the Mölndal, Sweden hospital and who were discharged to their own homes in the municipality of Mölndal during the period October 2008 to June 2010. Inclusion criteria were age 80 and older or age 65 to 79 with at least one chronic disease and dependence in at least one activity of daily living. Exclusion criteria included acute severe illness with immediate need (within 10 min) of assessment and treatment by a physician, severe cognitive impairment, and palliative care. The intention was that the study group would comprise a representative sample of frail elderly people at a high risk of future healthcare consumption. The study was approved by the regional Ethical Review Board in Gothenburg (ref. no 413–08).

### Procedure

Participants were consecutively recruited at the emergency department by nurses with geriatric competence during weekday daytimes (*n* = 144). Patients attending the emergency ward at other hours were recruited by either a visit to the wards or by letter, if discharged before recruitment (*n* = 17). Nurses informed the participants about the study both verbally and in writing. The information included a description of the study, how it would be conducted, and what was expected of persons who agreed to participate. Opportunities were provided for subjects to ask questions if anything was unclear. It was stressed, both in the verbal and the written information, that participation was voluntary. All participants signed a written consent form. Baseline data (from both interviews and assessments) were predominantly collected within a week of discharge. In three cases, however, data collection was postponed 1–2 weeks in consideration of participant strains, such as fatigue or illness.

### Data collection and measurements

All data were collected through study questionnaires during visits to participant homes by research assistants well trained in interviewing, assessing, and observing. Research assistants included occupational therapists, physiotherapists, and registered nurses; inter-rater reliability was tested to maximize assessment standardization. Study protocol meetings were held regularly throughout the study to reinforce the guidelines for the different outcome measurements in the questionnaire.

### Frailty indicators

Frailty was measured by Fried’s classical frailty indicators [7], with the addition of visual and cognitive impairment because of their high impact on disability. Indicators included: weakness, fatigue, weight loss, low physical activity, poor balance, low gait speed, visual impairment, and cognitive impairment. Cutoffs for weakness was a grip strength of less than 13 kg for women and 21 kg for men in the dominant hand, and 10 kg for women and 18 kg for men for the non-dominant hand (as measured by a hand dynamometer) [20]. Fatigue was noted if a participant answered “yes” to the question, “Have you suffered any general fatigue or tiredness over the last three months?” [21]. Weight loss was noted if a participant answered “yes” to the question, “Have you suffered from any weight loss over the last three months?” [21]. Low physical activity was defined as one to two walks per week or less. Poor balance involved a score of 47 or lower on the Berg balance scale [22]. Low gait speed was defined as walking 4 meters in 6.7 seconds or less [23]. Visual impairment was defined as a visual acuity of ≤0.5 in both eyes using the KM cart. Cognitive impairment was defined as <25 points in the Mini Mental State Examination [24]. Further details are available in the study protocol [18]. Subjects were determined as being frail when scores exceeded the cutoff value of three or more frailty indicators.

### Short screening instrument (FRESH-screening)

The FRESH-screening includes five short questions. The first four questions regarding mobility tiredness, fatigue, risk or fear of falling, and dependence in shopping were extracted from the “Continuum of care for frail elderly people” study questionnaire and were identified as early indicators of change in frailty by the research group. The four questions were as follows: 1) “Do you get tired when taking a short (15–20 min) walk outside?” (positive answers included both “yes,” and “can’t do it”) [25]; 2) “Have you suffered any general fatigue or tiredness over the last 3 months?” [21]. 3) “Have you fallen these last 3 months?” and “Are you afraid of falling?” (positive answers included “yes, a bit,” “yes,” and “yes, very afraid”); and 4) “Do you need assistance in either getting to the store, managing obstacles (such as staircases) to and from the store, or in choosing, paying for, or bringing home groceries?” [26]. The fifth question pertained to having had three or more emergency department (ED) visits over the last 12 months, which was considered clinically important by the healthcare service. The total number of healthcare visits was collected for each participant through registers. Subjects were considered to be at risk of frailty by answering “yes” to two or more of these five questions.

### Data analysis

The sensitivity, specificity, and predictive values were calculated to describe the probability of the five short screening questions being able to screen out those with frailty, as assessed by the eight frailty indicators.

A second analysis was performed to test the screening validity of the question pertaining to three or more ED visits during the last 12 months. Consequently, this fifth question was omitted in the second analysis.

Sensitivity was indicated by having two or more “yes” answers in the screening questions that correctly identified those with three or more frailty indicators. Specificity was indicated by having less than two “yes” answers that correctly identified those with less than three frailty indicators. The positive and negative predictive values generally give an assessment of the clinical usefulness of the test. The positive predictive value of this study was the proportion of persons correctly identified as being frail, while the negative predictive value was the proportion of those correctly identified as not being frail.

For screening purposes, a high sensitivity is commonly considered to be of high importance so as not to miss the identification of those in need of interventions. Additionally, a high specificity is also of importance to minimize unnecessary and costly CGAs. According to Evans [27] principles of screening, sensitivity and specificity should be similar [27]. A minimum of 80 % was set a priori for both values in this study. Furthermore, a screening test is according to Evans [27] considered to be of excellent clinical value if the sum of the sensitivity value and the positive predictive value percentages equal at least 150. In addition, the receiver operating characteristic (ROC) the area under curve (AUC) and its 95 % confidence interval (CI) was calculated. Higher AUC values were considered to demonstrate better discriminatory abilities as follows: excellent discrimination, AUC of ≥0.90; good discrimination, 0.80 ≤ AUC < 0.90; fair discrimination, 0.70 ≤ AUC < 0.80; and poor discrimination, AUC of < 0.70.

## Results

**Fig. 1:**
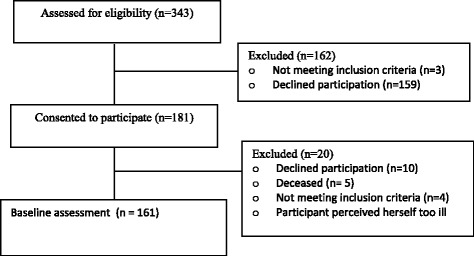
The flow of the participants from enrollment to baseline

During the inclusion period, 1445 elderly persons living in the municipality sought care at the emergency department. Of these persons, 343 met the inclusion criteria and were asked to participate; 181 persons consented to participate, 159 declined, and 3 were found to have an exclusionary cognitive impairment when further assessed. At the time of the baseline assessment, 20 participants were not assessed; 10 declined, 5 had deceased, 4 were excluded for not meeting study criteria, and 1 person perceived herself as too ill to continue participation. Thus, a final total of 161 subjects participated in the study (Fig. 1). For baseline characteristics, see Table 1.

**Table 1:** Baseline characteristics of participants

Characteristics	*n* = 161
Age mean (SD)	82 (5,5)
≥80 years n (%)	121 (76)
Female n (%)	89 (55)
Living alone	97 (60)
Academic education	22 (14)
Frail	117 (73)

### The five FRESH-screening questions (ED visits included) vs. frailty indicators

Sensitivity was high (84 %) but specificity was low (75 %) when comparing the five questions of the FRESH-screening against the eight frailty indicators (Table 2).

**Table 2:** Values for sensitivity, specificity, positive prediction, and negative prediction of the FRESH-screening compared with frailty indicators

	Five items screening	Four items screening
Sensitivity	98/117 = 84 %	95/117 = 81 %
Specificity	33/44 = 75 %	36/44 = 80 %
Positive prediction	98/109 = 90 %	95/103 = 92 %
Negative prediction	36/52 = 69 %	36/58 = 62 %
Sum of sensitivity and positive prediction	174	173

### The four FRESH-screening questions (ED visits excluded) vs. frailty indicators

Both sensitivity and specificity were high (81 % and 80 %, respectively) when comparing the four questions of the FRESH-screening against the eight frailty indicators. The sum of sensitivity and positive prediction percentages was 173 (81 % + 92 %), thus exceeding the 150 cutoff and demonstrating excellent clinical value. The AUC was 0.862, with a 95 % CI; 0.798 to 0.926.

## Discussion

The four-question FRESH-screening exhibited excellent clinical value in screening out frail older persons seeking acute care with a very high sensitivity and specificity, and the AUC (0.862) indicates a good discriminatory ability of the FRESH-screening instrument. The fifth question concerning multiple visits to the ED during the last 12 months was of no additional value when screening for frailty and may be omitted from the FRESH-screening instrument.

The question concerning three or more visits to the ED over the last year did not add to the validity of the screening instrument, indicating that being an older, frequent ED visitor is not equivalent to being frail. The inclusion of this question in the initial screening was initiated by the health service with the aim of rerouting these elderly persons to primary care and preventing readmission to the ED. International and national studies have found that frequent ED visitors of older ages have more acute illnesses and higher risk of hospitalization than occasional visitors; they also already have established contacts with primary care [28, 29]. Occasional visitors, however, have fewer preexisting primary care contacts [28]. One large study in Canada has shown that among those 65 years and older who seek care at the ED, 25 % leave the ED with no definite diagnosis, which suggests the possible presence of non-medical problems [30]. Thus, frequent visitors and occasional visitors may vary in their needs, demanding differing healthcare actions. Accordingly, it is valuable that the screening instrument identifies frail elderly persons irrespective of ED visit frequency.

This study’s results are promising compared with findings by Smets et al. [31] who recently tested four other common screening instruments. These instruments included the CGA (aCGA) [32], the vulnerable Elders Survey-13 (VES-13) [33], the Groningen Frailty Indicator (GFI) [2], and the Geriatric 8 (G8) [34]. None of the instruments showed both sensitivity and specificity above 80 % [31]. While the prevalence of frailty in the present study was higher (73 %) than in the study of Smets (50-60 %), the sensitivity and specificity values should not vary markedly as a function of prevalence [26]. The same four screening instruments were reviewed by Hamaker [35] to predict presence of impairments in CGA including papers in the context of geriatric oncology, with the same disappointing results. Hamaker [35] concludes the review by suggesting that developing targeted screening methods could be one way of increasing sensitivity and specificity. The FRESH-screening has been developed in close collaboration with multidisciplinary researchers and with multidisciplinary professionals in the ED, hospital, and community healthcare. The selection of included screening items was grounded both in research and in clinical experiences. This strategic collaboration may explain the excellent clinical value that has been recognized in our targeted screening instrument.

The sensitivity of 81 % means that the FRESH-screening did not detect 22 of the 117 who were frail; accordingly this affects the negative predictive value (62 %). The ultimate goal of any screening instrument is to detect all persons with a particular condition or disease, but realizing this goal among frail older persons may not be realistic. One way of upholding perceived good health despite frailty is having a positive view on one’s own life [36]. Thus, some frail older persons might not disclose frailty issues directly in a short screening session. As stated above sensitivity and specificity does not alter by prevalence rate. The predictive values though will change a lot by prevalence; the positive predictive value increases with high prevalence and the negative predictive value will decrease. Assuming all other factors remain constant the negative predictive value can vary from 95 % with prevalence of 25 % to 67 % with a prevalence of 75 % [16].

Because the FRESH-screening is appealingly easy to administer, one way to address non-detection is to implement the screening in other care settings outside of the ED, ensuring that those at risk of frailty will be detected early. Primary care settings are another example of clinical settings where frail older persons ought to be identified by screening. In one review, Pialoux [15] concludes that the potentially most promising screening instrument in primary care was the Tilburg Frailty indicator and the Share Frailty index. But the latter has not been tested for the age group over 75 years and requires a hand dynamometer. The Tilburg Frailty indicator takes a relatively long time to administer (14 min) [15]. In primary care, resources are scarce, and a user-friendly screening instrument such as the FRESH-screening could enhance screening implementation in practice. Further research on the FRESH-screening and its validity in primary care settings would be advantageous.

The specificity of 80 % means that the FRESH-screening falsely identified 8 persons as frail, who, according to the frailty indicators, were actually not frail. A low specificity would indicate that the healthcare system would need to perform further assessments, including measurements and questionnaires, at an additional cost of personnel.

Besides possessing excellent clinical value, the FRESH-screening has fewer questions than any of the aforementioned other screening instruments. Among the other instruments, the G8 has the fewest questions with eight questions, compared with four questions in the FRESH-screening. Next is the VES-13, consisting of 13 questions, while both aCGA and the GFI consist of 15 questions [30]. Recently, Kenig et al. [37] identified VES-13 as the best screening instrument, to predict postoperative morbidity and mortality among patients 65 years or older qualified for emergency abdominal surgery. Thirteen questions do not seem like many. Nevertheless, the less strenuous and time consuming a screening instrument is, the more likely it is that it will be implemented, especially at an emergency department. Overall, older persons arriving at an ED are more seriously ill, have more tests performed, and stay for longer times at the ED compared with younger persons [38]. According to the classical work by Wilson [17], screening instruments should not only be highly sensitive but also be as simple as possible, able to be carried out rapidly, and inexpensive. The four questions in the FRESH-screening take only a few minutes to administer, and our clinical experience is that both the older persons themselves and the personnel at the ED find the questions easy to answer, even in strenuous clinical situations that often present in EDs.

### Limitation of the study

Participants were only included consecutively during daytime which might hamper the representativeness. Among non-participants, reasons for declining participation in the study were both that health was too bad and too good. Non-participants were both in worse health and healthier than the participants. Therefore, it can be assumed that the participants in this study can be seen as a fairly representative sample of the frail older population attending an emergency department.

A potential limitation to the present study is that the participants did not answer the screening questions at the ED but rather in their homes afterwards. This procedure was chosen to be able to perform the screening simultaneously with the gold standard measurements. Scientifically, the optimal design would be to gather both the screening and the frailty measurements simultaneously at the ED, but gathering both simultaneously was considered too strenuous for the target group. Another limitation pertains to our choice of adding both visual acuity and cognition to Fried’s more classical frailty indicators [7]. Our rationale behind the addition of these items was that both visual impairment and memory problems have great impact on developing dependence in ADL; and the purpose of our instrument was to find those in early stages of developing ADL dependence [9].

## Conclusion

Despite these limitations, however, this study shows the FRESH-screening to be of excellent clinical value. Additionally, the clinical experience is that the instrument is simple and rapid to use, takes only a few minutes to administer, and requires minimal energy input by older Further studies are needed to test the FRESH-screening’s potential in other settings such as primary care.

## Abbreviations

ADL, activities of daily living; AUC, area under the curve; CGA, comprehensive geriatric assessment; CI, confidence interval; ED, emergency department; ROC: receiver operating characteristic
